# Small bowel obstruction SBO after TAPP repair caused by a self-anchoring barbed suture device for peritoneal closure: case report

**DOI:** 10.1093/jscr/rjy165

**Published:** 2018-07-20

**Authors:** Eugenio M Tagliaferri, Sheng L Wong Tavara, Jacky L Abad de Jesus, Heinrich Bergmann, Sebastian Hammans, Christoph M Seidlmayer

**Affiliations:** Department of Surgery, St. Bonifatius Hospital, Lingen, Germany

## Abstract

A 50-year-old man underwent laparoscopic hernia repair for a groin hernia, presenting acute abdominal pain and bowel obstruction syndrome 1 day post surgery. Diagnostic laparoscopy was performed at postoperative the day after the hernioplasty and a volvulus was found. The residual end of the barbed V-LOC adopted in the peritoneal closure was incidentally hooked to the mesentery and caused a small bowel obstruction as a volvulus. The redundant V-LOC strand was released and cut superficial to the peritoneum. A detorsion of volvulus was preformed. Neither bowel ischemia nor significant bowel injury was noted. The following day he was discharged without complication. The residual ‘free’ barbel suture in the peritoneal cavity invited adhesion formations and subsequently the Bowel obstruction.

## INTRODUCTION

Laparoscopic trans-abdominal hernia repair is commonly preformed nowadays due to its advantages as a minimally invasive approach with less postoperative discomfort and a shorter hospital stay [1].

To facilitate the closure of the peritoneum, barbed suture is often applied since no knots have to be tied.

In recent years there has been an increasing uptake in the use of barbed suture, particularly in minimally invasive and laparoscopic procedures as they may ease the surgical procedure.

The V-Loc™ suture consists of a monofilament with tiny barbs cut into the length of the surface, self-anchoring to the tissue. This self-anchoring capacity eliminates the need for surgical knots and laparoscopic procedure becomes easier by reducing operating time [2].

However, as it has been demonstrated in several recent case reports [3, 4] a potential downside to this practice and to the use of such suture material is that exposed suture barbs may catch on adjacent small bowel, mesentery or omentum leading to serosa injury, obstruction or volvulus.

Postoperative small bowel obstruction (SBO) is common and has often been linked to the presence of foreign materials, particularly surgical mesh [5]. Here we report one more case of SBO subsequently requiring surgical re-intervention that was directly related to the use of barbed sutures. In this case these complications arose from adherence of the suture’s distal end to small bowel and mesentery.

This case report describes an early complication of entrapment and volvulus of the small bowel following the use of barbed suture in TAPP repair.

## PRESENTATION OF CASE

A 50-year-old man is presented as an emergency derived from another hospital with several abdominal pain and obstipation.

One day before admission a TAPP procedure was performed with peritoneal closure using a V-LOC barbed suture.

On the first day postoperative the patient developed diffuse abdominal pain and distension with associated vomiting and intolerance of feed.

**Figure 1:**
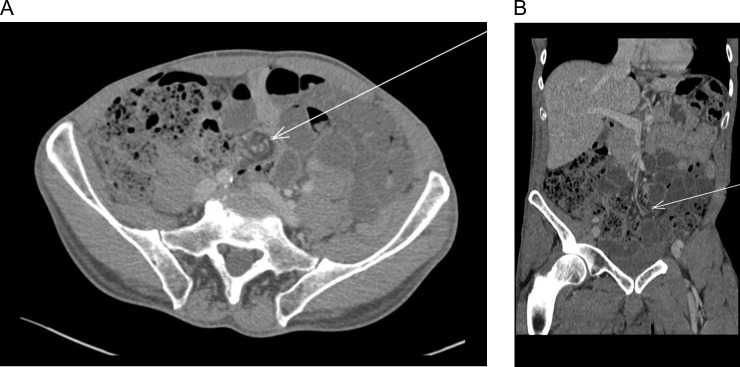
(**A**) Abdominal computed tomography (axial) revealed dilation of small intestine with a rotation of the mesentery around the mesenteric vessels (whirl sign) (arrow). (**B**) Abdominal computed tomography (coronal) revealed dilation of small intestine with a rotation of the mesentery around the mesenteric vessels (whirl sign) (arrow).

An abdominal computerized tomography (CT) demonstrated SBO with suspected volvulus (Fig. 1).

He was returned to theater for laparascopic exploration.

**Figure 2:**
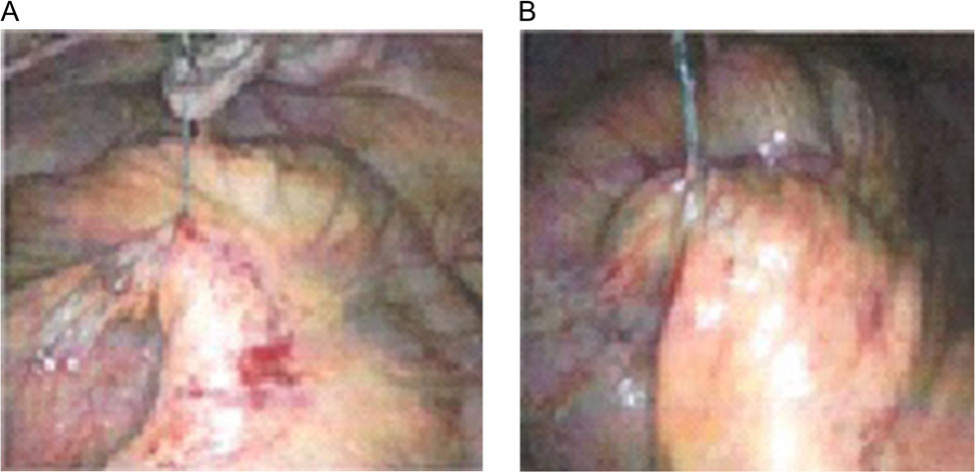
(**A**) Laparoscopic exploration revealed to barbed suture exposed from the peritoneum and wrapped in mesentery. (**B**) Laparoscopic exploration revealed to small intestine volvulus.

SBO was noted secondary to the cut end V-LOC suture, which had become integrated into the small bowel mesenterium creating a volvulus with associated ischemia (Fig. 2).

Release of adherent suture and derotation resulted in a good re-perfusion and no resection was required.

The patient recovered well and was discharged on the first postoperative day when the examination revealed a soft abdomen and he was able to tolerate food and passed bowel motion.

## DISCUSSION

This is a case of SBO by barbed suture used for peritoneal closure. Acute abdominal pain and small bowel volvulus occurred 1 day after surgery therefore in the same day the patient received emergent laparoscopy to release the barbed suture, and derotation of the small bowel was performed without complications.

The mechanism of SBO documented in this case is comparable to these described in the literature [6].

The V-Loc™ wire can be used for closure of the peritoneum during the TAPP procedure. Due to its barbed configuration, a gapless and continuous suturing of the peritoneum without laparoscopic knotting is fast and easily accomplished [7].

Where the cut end of the barbed suture is left long, it may become firmly attached to underlying small bowel or mesentery producing kinking and a transition point of obstruction. Torsion of the small bowel around this point of fixation may result in volvulus and where mesenteric blood flow is impeded ischemia may occur.

Apart from TAPP the use of the barbed suture in the closure of the peritoneum had also been implicated to cause bowel complications. Lee and Wong [8] reported a case of SBO following peritoneal closure at laparoscopic myomectomy using a barbed suture and the patient was presented with bowel obstruction 6 weeks after the procedure. SBO could present itself as early as 1 day following the use of barbed suture in closure of peritoneum after laparoscopic ventral mesh rectopexy [9]. Segura-Sampedro *et al.* [10] performed a search of electronic database and revealed up to 15 cases of SBO complicating laparoscopic pelvic surgery following the use of barbed suture in laparoscopic operations and reported two cases in his practice.

The common feature of all reported cases was that the ends of the barbed suture was left in the abdominal cavity in contact with the small intestine.

## CONCLUSIONS

These cases highlight that although barbed sutures provide an attractive mean to allow easier and faster laparoscopic suturing, they should be used carefully. The residual ‘free’ barbed suture in the peritoneal cavity invites adhesion formations and subsequently the bowel obstruction. We suggest not to leave the free V-LOC suture in the peritoneal cavity. In the case of SBO complicating the early period after barbed suture it is important to consider barbed suture entanglement as a potential etiology for this condition.

## References

[1] Bittner R, Arregui ME, Bisgaard T, Dudai M, Ferzli GS, Fitzgibbons RJ, et al Guidelines for laparoscopic (TAPP) and endoscopic (TEP) treatment of inguinal hernia. Surg Endosc 2011;25:2773–2843. 21751060 10.1007/s00464-011-1799-6 PMC3160575

[2] Nemeck E, Negrin L, Beran C, Nemecek R, Hollinsky C The aplication of the V-Loc closure device for gastrointestinal suture: a preliminary study. Surg Endosc 2013;27:3830–4. 23644839 10.1007/s00464-013-2982-8

[3] Filser J, Reibetanz J, Krajinovic K, Germer CT, Dietz UA, Seyfried F Small bowel volvulus after transabdominal preperitoneal hernia repair due to improper use of V-Loc™ barbed absorbable wire—do we always ‘read the instructions first’? Int J Surg Case Rep 2015;8:193–5. 10.1016/j.ijscr.2015.02.020 PMC4353972 25704567

[4] Chen H, Hong M-K, Ding D-C Acute small bowel ostruction by barbed suture on second day after laparoscopic hysterosacropexie: a case report and literatur review. Taiwan J Obstet Gynecol 2017;56:247–9. 28420518 10.1016/j.tjog.2016.03.008

[5] Luijendijk RW, de Lange DC, Wauters CC, Hop WC, Duron JJ, Pailler JL, et al Foreign materialin postoperative adhesions. Ann Surg 1996;223:242–8. 8604903 10.1097/00000658-199603000-00003 PMC1235111

[6] Buchs NC, Ostermann S, Hauser J, Roche B, Iselin CE, Morel P Intestinal obstruction following use of laparoscopic barbed suture: a new complication with new material? Minim Invasive Ther Allied Technol 2012;21:369–71. 22145693 10.3109/13645706.2011.638643

[7] Ovesen RJ, Friis-Andersen H Ileus caused by V-loc sutures. Ugeskr Laeger 2014;176 (25A). pii: V03130165. 25497618

[8] Lee ET, Wong FW Small bowel obstruction from barbed suture following laparoscopic myomectomy—a case report. Int J Surg Case Rep 2015;16:146–9. 26454501 10.1016/j.ijscr.2015.09.039 PMC4643471

[9] Vasudevan SP, Dworkin MJ Small bowel obstruction following laparoscopic ventral mesh rectopexy. Colorectal Disease 2013;15:1543–4. 24034476 10.1111/codi.12402

[10] Segura-Sampedro JJ, Ashrafian H, Navarro-Sánchez A, Jenkins JT, Morales-Conde S, Martínez-Isla A Small bowel obstruction due to laparoscopic barbed sutures: an unknown complication? Original papers. Rev Esp Enferm Dig (Madrid) 2015;107:677–80. 10.17235/reed.2015.3863/2015 26541657

